# Sex differences in response of the bovine embryo to colony-stimulating factor 2

**DOI:** 10.1530/REP-16-0336

**Published:** 2016-10-20

**Authors:** Luiz G B Siqueira, Peter J Hansen

**Affiliations:** 1 Department of Animal SciencesD.H. Barron Reproductive and Perinatal Biology Research Program, and Genetics Institute, University of Florida, Gainesville, Florida, USA; 2 Embrapa Gado de LeiteJuiz de Fora, MG, Brazil

## Abstract

We tested whether gene expression of the bovine morula is modified by CSF2 in a sex-dependent manner and if sex determines the effect of CSF2 on competence of embryos to become blastocysts. Embryos were produced in vitro using X- or Y-sorted semen and treated at Day 5 of culture with 10 ng/mL bovine CSF2 or control. In experiment 1, morulae were collected at Day 6 and biological replicates (*n* = 8) were evaluated for transcript abundance of 90 genes by RT-qPCR using the Fluidigm Delta Gene assay. Expression of more than one-third (33 of 90) of genes examined was affected by sex. The effect of CSF2 on gene expression was modified by sex (*P* < 0.05) for five genes (*DDX3Y/DDX3X-like*, *NANOG*, *MYF6*, *POU5F1* and *RIPK3*) and tended (*P* < 0.10) to be modified by sex for five other genes (*DAPK1*, *HOXA5*, *PPP2R3A*, *PTEN* and *TNFSF8*). In experiment 2, embryos were treated at Day 5 with control or CSF2 and blastocysts were collected at Day 7 for immunolabeling to determine the number of inner cell mass (ICM) and trophectoderm (TE) cells. CSF2 increased the percent of putative zygotes that became blastocysts for females, but did not affect the development of males. There was no effect of CSF2 or interaction of CSF2 with sex on the total number of blastomeres in blastocysts or in the number of inner cell mass or trophectoderm cells. In conclusion, CSF2 exerted divergent responses on gene expression and development of female and male embryos. These results are evidence of sexually dimorphic responses of the preimplantation embryo to this embryokine.

## Introduction

Optimal development of the preimplantation embryo depends upon signals from the maternal reproductive tract. Alterations in the maternal environment during the preimplantation period can not only alter competence of the embryo to establish pregnancy (Holm *et al*. 1994, Siqueira *et al*. 2009, Farin *et al*. 2010) but also alter postnatal function of the offspring (Kwong *et al*. 2000, Watkins *et al*. 2007, Fleming *et al*. 2015). One of the characteristics of developmental programming of postnatal function by maternal inputs during the preimplantation period is that alterations in phenotype often are different for male offspring than female offspring (Hansen *et al*. 2016). For example, mice derived by in vitro fertilization had altered postnatal growth and exhibited glucose intolerance, but these effects were only seen in males (Donjacour *et al*. 2014). In sheep, restriction of vitamin B and methionine supply in the maternal diet during the periconceptional period led to changes in the phenotype of adult male offspring, which were heavier, fatter and insulin-resistant, and had elevated blood pressure. These effects were not observed in the female offspring (Sinclair *et al*. 2007).

One possible explanation for the phenomenon of sexual dimorphism in developmental programming is that certain maternal embryokines exert different actions on male embryos than on female embryos. Indeed, sexual dimorphism is already apparent in the eight-cell mouse embryo (Lowe *et al*. 2015) and by the morula stage in bovine (Denicol *et al*. 2015). In the bovine blastocyst, one-third of expressed genes are differentially expressed between male and female embryos (Bermejo-Alvarez *et al*. 2010). The epigenome of bovine embryos is also affected by sex and this effect varies by stage of development; based on labeling of 5-methyl cytosine (a marker of DNA methylation) female embryos had more DNA methylation at the 6–8 cell stage compared with males, whereas the opposite was true at the blastocyst stage (Dobbs *et al*. 2013*a*). In addition, female bovine blastocysts had a greater proportion of cells that were apoptotic than males, particularly for inner cell mass (ICM) compared with trophectoderm (TE) (Ghys *et al*. 2016).

One embryokine that affects male embryos differently than female embryos is colony-stimulating factor 2 (CSF2). Expressed in the oviduct and endometrium in a variety of species, including cattle (Tremellen *et al*. 1998, de Moraes *et al*. 1999, Robertson 2007), CSF2 can increase the proportion of cultured embryos that develop to the blastocyst stage in cattle (Loureiro *et al*. 2009), humans (Sjöblom *et al*. 1999, Ziebe *et al*. 2013), mice (Sjöblom *et al*. 2005) and pigs (Kwak *et al*. 2012). Moreover, CSF2 increased survival of embryos after transfer into recipients in cattle (Loureiro *et al*. 2009, Denicol *et al*. 2014), mice (Sjöblom *et al*. 2005) and humans (Ziebe *et al*. 2013), affected embryo gene expression in several species (Behr *et al*. 2005, Chin *et al*. 2009, Sferruzzi-Perri *et al*. 2009, Loureiro *et al*. 2011), inhibited apoptosis in the bovine (Loureiro *et al*. 2011), and altered the number of cells in the ICM of the bovine blastocyst (Loureiro *et al*. 2009) or TE of the pig blastocyst (Kwak *et al*. 2012).

Response to CSF2, however, is characterized by sexual dimorphism. In the cow, treatment with CSF2 from Day 5 to 7 after fertilization caused different effects on the process of trophoblast elongation for male embryos at Day 15 of gestation than for female embryos (Dobbs *et al*. 2014). For male embryos, CSF2 increased elongation of the conceptus and accumulation of the antiluteolytic molecule IFNT in the uterus. For female embryos, the opposite occurred, with CSF2 decreasing elongation and accumulation of IFNT. In addition, CSF2 affected gene expression and DNA methylation of the trophoblast in distinct ways for male and female embryos. These findings are indicative that CSF2 acted on the male embryo at Day 5–7 differently than it did on the female embryo and in a way that resulted in sexually dimorphic responses later in pregnancy. This phenomenon is not limited to cattle. In the mouse, treatment of cultured embryos with CSF2 changed phenotype of the resultant offspring during the postnatal period, with males being affected differently than females (Sjöblom *et al*. 2005).

The overall objective of the current study was to test the hypothesis that sexually dimorphic actions of CSF2 on the bovine preimplantation embryo are evident at the morula and blastocyst stages of development. This hypothesis was tested by examining several characteristics of preimplantation development. First, it was tested whether alteration of gene expression by CSF2 depended on sex. By the morula stage, the time examined here, 128 genes were identified whose expression differed between male and female embryos by >1.5 fold (Denicol *et al*. 2015). Since CSF2 has been reported to either increase or decrease the percent of embryos developing to the blastocyst stage of development, depending on the overall level of development (Dobbs *et al*. 2013*b*), we tested the hypothesis that embryo sex would affect actions of CSF2 on the competence of the embryo to become a blastocyst. Finally, it was tested whether the effects of CSF2 on differentiation of the blastocyst into TE or ICM depended on sex.

## Methods

### Experimental design

Two experiments were performed to investigate the consequences of CSF2 on preimplantation development of female and male embryos. Experiment 1 was designed to test the hypothesis that the effect of CSF2 on gene expression at the morula stage (Day 6 of development) was modified by sex. The experiment involved a randomized complete design with a 2 × 2 arrangement of treatments with main effects of treatment (control vs CSF2) and embryo sex (female vs male). Experiment 2 used a similar design to test the hypothesis that the effects of CSF2 on development of embryos to the blastocyst stage at Day 7 of development and lineage commitment in the resultant blastocysts depended on sex.

### In vitro production of embryos

Embryos were produced in vitro following the procedures described previously (Dobbs *et al*. 2013*b*, Denicol *et al*. 2015). All chemicals were obtained from Sigma-Aldrich or Thermo Fisher unless otherwise stated. Oocyte washing medium (BoviPRO) contained salts, bicarbonate, HEPES, DL-lactic acid, and bovine serum albumin (BSA) and was purchased from MOFA Global (Verona, WI, USA). Oocyte maturation medium consisted of Tissue Culture Medium-199 with Earle’s salts (Gibco) supplemented with 10% (v/v) bovine steer serum (Bioreclamation IVT, Baltimore, MD, USA), 2 µg/mL estradiol 17-β, 20 µg/mL porcine follicle stimulating hormone (Bioniche Animal Health, Athens, GA, USA), 22 µg/mL sodium pyruvate, 50 µg/mL gentamicin sulfate, and 1 mM glutamine. Tyrode’s albumin lactate pyruvate (TALP) solutions including HEPES-TALP, Sperm-TALP and IVF-TALP were prepared as described by (Parrish *et al*. 1986) by modifying base solutions provided as a custom preparation from Caisson (Smithfield, UT, USA). Embryos were cultured in a serum-free culture medium termed SOF-BE2 (synthetic oviduct fluid – bovine embryo 2); (Kannampuzha-Francis *et al*. 2016). Straws of X- and Y-sorted semen were from commercially available beef sires and purchased from ABS Global (De Forest, WI, USA) and Genex Cooperative Inc. (Shawano, WI, USA).

Cumulus–oocyte complexes (COC) were retrieved from follicles 2–8 mm in diameter by scoring the surface of ovaries collected from a local abattoir with a scalpel followed by vigorously mixing with BoviPRO oocyte washing medium. Those COC containing at least three layers of compact cumulus cells and a homogeneous cytoplasm were selected for in vitro maturation, fertilization and culture. After washing twice in BoviPRO, groups of 10 COCs were transferred to 50 µL drops of oocyte maturation medium, covered with mineral oil and matured for 22–24 h at 38.5°C, 5% CO_2_ in a humidified atmosphere.

After maturation, groups of 30 COC were washed three times in HEPES-TALP and transferred to fertilization drops covered with mineral oil. Each drop contained 60 µL IVF-TALP and 3.5 µL of a PHE solution (0.05 mM penicillamine, 0.25 mM hypotaurine, and 25 µM epinephrine). Drops containing COC were fertilized with 20 µL of X- or Y-sorted sperm purified using the Puresperm 40/80 gradient column (Nidacon International AB, Mölndal, Sweden). A total of nine Angus and Simmental bulls were used in each of the two experiments. For each replicate (i.e., a set of COC collected on a specific day), a pool of 2–3 bulls randomly chosen from among the 9 bulls was used for fertilization. For each replicate, X and Y-sorted semen came from the same bulls. The sperm purification procedure consisted of centrifugation (2600 ***g*** for 5 min) in 2.0 mL microcentifuge tubes of 0.25 mL sperm over two layers of 200 µL of Puresperm (top layer Puresperm40, bottom layer Puresperm80). The pellet representing the bottom 100 µL was transferred to a new microcentrifuge tube, washed in 1000 µL of IVF-TALP that had been pre-equilibrated at 38.5°C under 5% CO_2_, and centrifuged at 600***g*** for 3 min. The final concentration in the fertilization drop was approximately 2 × 10^6^ sperm/mL.

After 18 h of co-incubation of gametes at 38.5°C and 5% CO_2_ in humidified air, putative zygotes (i.e., oocytes exposed to sperm) were removed from fertilization drops and denuded of cumulus cells by vortexing for 5 min in 100 µL hyaluronidase stock solution (10,000 U/mL) diluted in 600 µL HEPES-TALP. Putative zygotes were then washed twice in HEPES-TALP and a third time in SOF-BE2. They were then placed in culture in groups of 25–30 in 63 µL microdrops of SOF-BE2, covered with mineral oil and incubated at 38.5°C in a humidified atmosphere of 5% CO_2_ (v/v), 5% O_2_ (v/v) and 90% N_2_ (v/v). Cleavage was assessed on Day 3 after insemination (72 h post-insemination, hpi). Treatments (control or CSF2) were applied to culture drops on Day 5 (120 hpi) by adding 7 µL of vehicle (Dulbecco’s phosphate-buffered saline (DPBS) containing 1% (w/v) BSA) or 7 µL of a 100 ng/mL CSF2 solution. The final concentration of CSF2 in the 70 µL culture drops was 10 ng/mL. This concentration of CSF was used because it has been shown to affect several characteristics of the bovine embryo (Loureiro *et al*. 2009, 2011; Dobbs *et al*. 2013*b*; Denicol *et al*. 2014). The recombinant bovine CSF2 was either obtained as a gift from CIBA-GEIGY (Basle, Switzerland) or was purchased from Kingfisher Biotech (St. Paul, MN, USA). Both preparations had similar bioactivity as determined by upregulation of *NOS2* mRNA in bovine monocyte cultures using methods described elsewhere (Merriman *et al*. 2015). Embryos were cultured until Day 6 (144 hpi) (Experiment 1), for collection of morulae for gene expression, or Day 7 (Experiment 2), when blastocysts were counted and collected for analysis of numbers of inner cell mass (ICM) and trophectoderm (TE) cells.

### Experiment 1: effects of CSF2 on gene expression in male and female morulae

#### Collection and processing of morulae

Morulae (*n* = 1600) were collected at Day 6 after insemination (144 hpi) from cultures of 4850 oocytes fertilized with X- or Y-sorted semen in 22 replicates. Only morulae that were not undergoing morphological signs of degeneration were selected for analysis. Immediately after collection, morulae were washed three times in 50 µL droplets of diethylpyrocarbonate (DEPC)-treated DPBS containing 0.1% (w/v) PVP and incubated with DEPC-treated 2× DPBS–0.1% (w/v) protease from *Streptomyces griseus* for zona pellucida removal. Once zonae could not be seen surrounding the embryo (10–15 min incubation in protease), zona-free morulae were washed three more times in DPBS–PVP, transferred in a 5 µL suspension of DEPC-treated DPBS–PVP to autoclaved 2.0 mL RNase/DNase-free microcentrifuge tubes (Corning), and snap frozen in liquid nitrogen. Embryos within each in vitro fertilization (IVF) procedure were snap frozen together and then combined with embryos from other IVF procedures to form pools of 50 morulae, which was defined as a biological replicate. There were a total of eight replicates per treatment. Samples were stored at −80°C until RNA extraction.

#### RNA extraction

RNA from each of the eight pools of 50 frozen-thawed morulae was extracted using the Qiagen RNeasy Micro Kit (Qiagen) following the manufacturer’s instructions. The RNA isolation procedure included DNase treatment. Elution of RNA was performed in a volume of 20 µL (10 + 10 µL). Eluted RNA was evaluated for integrity (RIN) using the TapeStation 2200 machine (Agilent Technologies). Concentrations of RNA ranged from 254 to 1010 pg/µL and RIN numbers from 6.3 to 10. Extracted RNA was stored at −80°C until further analysis by qPCR.

#### PCR primers

A set of 96 PCR primers corresponding to 92 genes of interest plus four housekeeping genes (*ACTB*, *GAPDH*, *SDHA* and *YWHAZ*) was prepared by Fluidigm for the Fluidigm Delta Gene assay (Fluidigm Co., San Francisco, CA, USA). Among the collection of genes of interest there were 49 genes previously demonstrated to be regulated by CSF2 (Loureiro *et al*. 2011), 13 genes regulated by embryo sex (Denicol *et al*. 2015), and 30 genes important for embryonic development, cellular differentiation and involved in apoptosis. Details of genes and primers are in Supplemental File S1, see section on supplementary data given at the end of this article.

A qualification run was performed to validate the primers for all 96 genes using four control samples of RNA. The qualifications were performed with 8-point, two-fold dilution series (replicated three times), and linear relationships between RNA amount and Ct values were analyzed as described previously (Dominguez *et al*. 2013). Briefly, an initial 3.5 µL of each sample was pre-amplified in a 10 µL reaction for 18 cycles followed by exonuclease I treatment. Then, each sample was diluted in two-fold serial dilutions for a total of eight dilutions (D1–D8) and three replicates per sample. A water sample was included as a non-template control (NTC). The quality control criteria for passing a primer were an *R*^2^≥0.97, efficiency of 0.8–1.3 and a slope of −3.92 to −2.76. Primers targeting two genes (*CACNA1G* and *CNR2*) failed to pass the quality control in the qualification run and were excluded from analysis. Thus, the final set of genes analyzed was composed of 90 genes of interest plus the four housekeeping genes.

#### Reverse transcription and cDNA synthesis

RNA from morulae was subjected to reverse transcription and cDNA synthesis using the Cells Direct Kit (Life Technologies), per manufacturer's instructions. A total of 18 cDNA synthesis cycles were performed on 500 pg RNA, followed by exonuclease I treatment and loading into the microfluidic chip (1:4 dilution). Each sample was run in technical triplicates, except for one sample of female embryos treated with CSF2, which was run in technical duplicates so that a no template control (NTC) could be included on the microfluidic chip.

#### Analysis of gene expression

Gene expression was analyzed by the Fluidigm qPCR procedure, using the microfluidic device Biomark HD system. Primer–probe sets and samples were transferred to an integrated fluidic circuits (IFC) plate and loaded into an automated controller that prepares the nanoliter reactions. The IFC plate was ran on the Biomark machine, which uses a thermal cycler for real-time quantitative PCR. The software Fluidigm Real-Time PCR Analysis was used to establish standard curves and calculate cycle threshold (Ct) values.

Forty cycles of PCR were performed, using the 96.96 dynamic array IFC (microfluidic chip) developed by the manufacturer. Non-detectable expression was considered to be a Ct of 27. ΔCt values were calculated relative to the geometric mean of the four housekeeping genes in the 96-gene set. Fold changes were calculated as 2^−ΔCt^. Note that ΔCt was calculated instead of ΔΔCt to allow discrimination between genes regarding the magnitude of expression relative to housekeeping genes. Gene expression was analyzed in a total of 32 samples (female-control; female-CSF2, male-control; male-CSF2) from eight biological replicates consisting of pools of 50 morulae each.

#### Statistical analysis

Statistical analysis was performed using SAS software (version 9.3: SAS Institute Inc., Cary, NC, USA). Outcome variables were development to the morula stage (percent of total putative zygotes) and ΔCt of genes evaluated by qPCR. Real-time qPCR data (ΔCt values) were analyzed using the PROC MIXED procedure for the effects of sex (female vs male), treatment (control vs CSF2) and their interaction. Replicate was included in the model as a random variable. If the *P* < 0.10) for a sex or treatment effect, pairwise differences of least-squares means were examined to determine the treatment effects (control vs CSF2) within each sex using the PDIFF statement of SAS. Gene expression data represent least-squares means ± s.e.m. of fold change relative to the housekeeping genes.

### Experiment 2: sex-specific effects of CSF2 on embryonic development and blastocyst cell number and lineage allocation

#### Embryo competence to reach the blastocyst stage

Embryos fertilized with X- or Y-sorted sperm were evaluated for development on Day 7 (168 hpi). A total of nine replicates with 351–427 putative zygotes per treatment group (total = 1612) were placed in culture. Proportions of total putative zygotes and cleaved embryos becoming blastocysts at Day 7 were recorded for each sex (female vs male) and treatment (control vs CSF2). An embryo was considered a blastocyst if a fully formed blastocoel was present (both blastocysts and expanded blastocysts).

#### Blastocyst immunolabeling and differential cell counting

Day 7 blastocysts were collected and fixed before staining for immunofluorescence (*n* = 9 replicates and 210 blastocysts). The numbers of embryos providing data on cell number were: female-control (*n* = 46), female-CSF2 (*n* = 51), male-control (*n* = 57) and male-CSF2 (*n* = 56). Embryos were washed three times in DPBS plus 0.1% (w/v) polyvinylpyrrolidone (DPBS–PVP), fixed in 4% (w/v) paraformaldehyde for 15 min at room temperature, and then washed three more times in DPBS–PVP. For immunostaining, fixed blastocysts were first permeabilized by incubation for 20 min in 0.5% (v/v) Triton X-100 in DPBS, followed by three washes in wash buffer (DPBS containing 0.1% (v/v) Tween 20 and 0.1% (w/v) BSA), and incubation in blocking buffer (DPBS plus 5% (w/v) BSA) for 1 h at room temperature. Embryos were then incubated overnight at 4°C in the darkness with a primary mouse monoclonal antibody against the transcription factor CDX2 (Ready-to-use CDX2-88; Biogenex, Fremont, CA, USA), a TE cell marker. As a negative control (two embryos per procedure), anti-CDX2 was replaced with 1 µg/mL mouse IgG (Sigma-Aldrich) diluted in antibody buffer (0.1% (v/v) Tween 20 and 1% (w/v) BSA in DPBS). Embryos were then washed three times in wash buffer and incubated with 2 µg/mL fluorescein isothiocyanate (FITC)-conjugated goat anti-mouse IgG heavy and light chain (Abcam) diluted in antibody buffer for 1 h at room temperature in the darkness. After three washes in wash buffer, embryos were incubated with a nuclear probe (1 µg/mL, Hoechst 33342; Thermo Fisher Scientific) diluted in DPBS–PVP for 15 min at room temperature in the darkness. Embryos were washed once in DPBS–PVP and mounted onto a glass slide with an anti-fade solution (SlowFade Gold Antifade Mountant, Molecular Probes, Life Technologies) and covered with a cover slip.

Slides were visualized using an epifluorescence microscope (Zeiss Axioplan 2, Zeiss) with 40× objective (400× magnification) using Zeiss filters set 02 (DAPI filter) and set 03 (FITC filter). Digital images of each embryo were obtained using the software AxioVision (v. 4.8.2; Zeiss) and a high-resolution black and white Zeiss Axiocam MRM digital camera. Differential cell counting was performed using the imageJ software (http://www.imagej.nih.gov). The total number of cells in each embryo was assessed by counting all nuclei labeled positive for Hoechst 33342. Cells labeled with anti-CDX2 were considered TE cells and the number of ICM cells was calculated by subtraction of number of TE cells from the total number of cells.

#### Statistical analysis

Statistical analysis was performed using SAS software (version 9.3: SAS Institute Inc., Cary, NC, USA) and the outcome variables were development to the blastocyst stage (percent of total putative zygotes and percent of cleaved embryos), total number of cells in the blastocyst, TE cell number, and ICM cell number. Proportions (percent cleavage, total putative zygotes and cleaved embryos becoming blastocysts) were analyzed by PROC GLM procedure of SAS using rates of development within each replicate as the experimental unit. Analysis of blastocyst cell numbers (ICM, TE and TE:ICM ratio) was also performed using the PROC GLM; data represent least-squares means ± s.e.m. Replicate was considered random and included in the model. Other main effects were considered fixed. Statistical significance was determined based on a *P* value <0.05 and tendency based on *P* values between 0.05 and 0.10. If a tendency was detected, the PDIFF statement of SAS was performed to determine differences among least-square means within the same sex.

## Results

### Experiment 1: effects of CSF2 on gene expression of the bovine morula in female and male embryos

**Figure 1:**
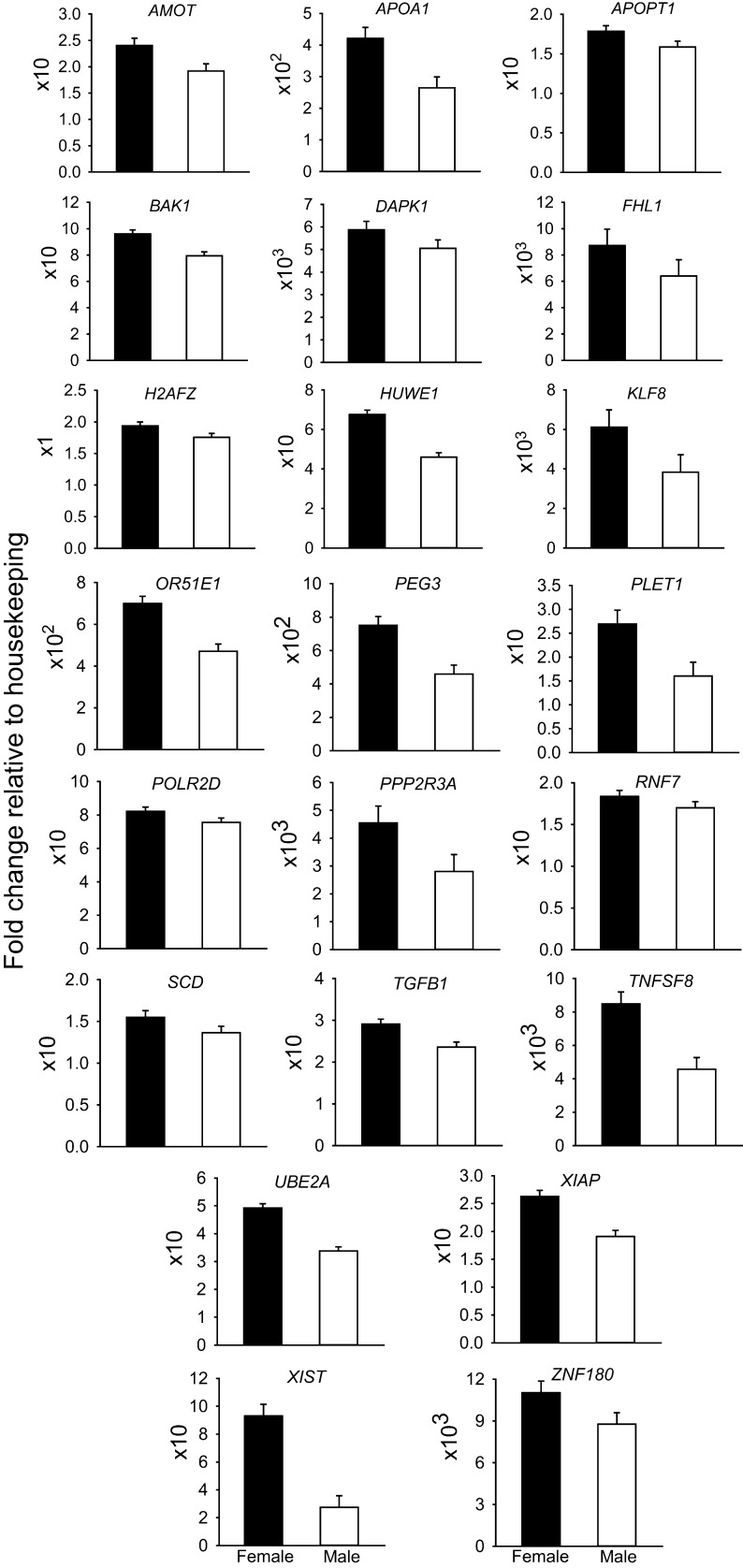
Genes upregulated in female embryos. Data represent the fold change of level of expression relative to housekeeping genes. Fold change was multiplied by the factor shown on the Y axis for ease in graphing. Results are least-squares means ± s.e.m. of eight biological replicates of 50 pooled morulae each. The effect of sex was *P* < 0.05 for all genes. Note that the following genes are X-linked: *AMOT*, *FHL1*, *HUWE1*, *KLF8*, *UBE2A*, *XIAP*, *XIST*.

**Figure 2:**
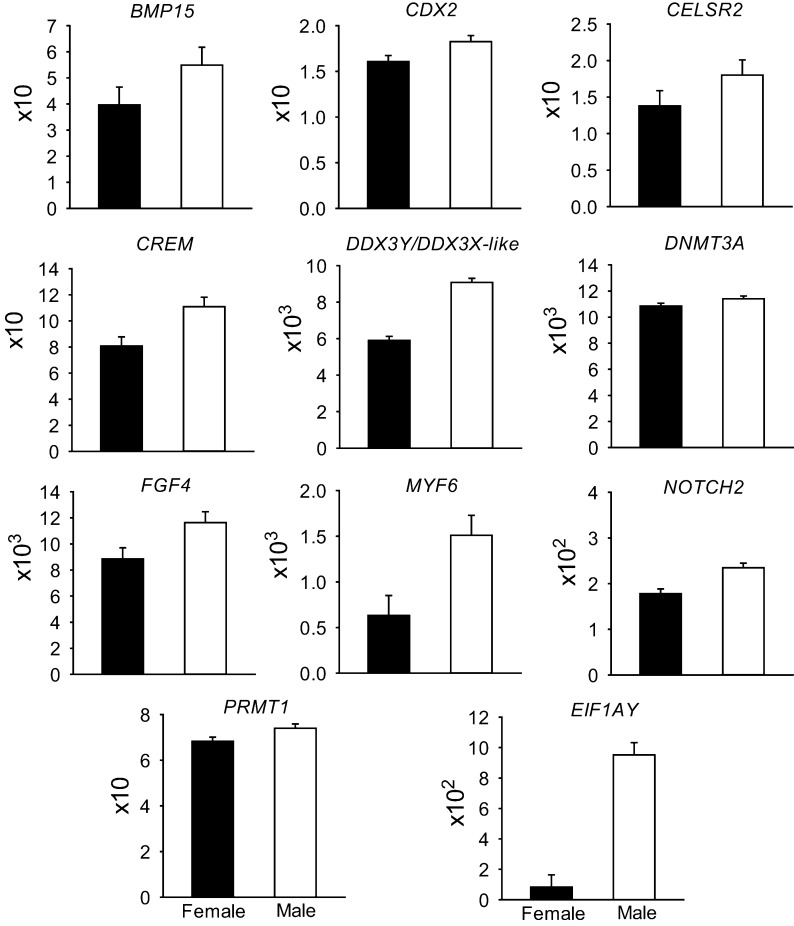
Genes upregulated in male embryos. Data represent the fold change of level of expression relative to housekeeping genes. Fold change was multiplied by the factor shown on the *Y* axis for ease in graphing. Results are least-squares means ± s.e.m. of eight biological replicates of 50 pooled morulae each. The effect of sex was *P* < 0.03 for all genes. Note that *DDX3Y*, *EIF1AY* are Y-linked and *BMP15* is X-linked. The primers for *DDX3Y* hybridize with *DDX3X-like*.

Least-squares means for all genes examined are presented in Supplemental File S1. Expression of 33 genes was significantly affected by sex (*P* < 0.05), with 22 genes upregulated in females (Fig. 1) and 11 upregulated in males (Fig. 2). Of the 22 genes upregulated in females, seven are located in the X chromosome (*AMOT*, *FHL1*, *HUWE1*, *KLF8*, *UBE2A*, *XIAP*, *XIST*). Of the 11 genes upregulated in males, two are located on the Y-chromosome (*DDX3Y*, *EIF1AY*) and another is on the X chromosome (*BMP15*). The primers for *DDX3Y* also crossreacted 100% with *DDX3X-like* (LOC107131212) located on the X chromosome. Note that the set of genes whose expression was regulated by sex includes 9 of 12 genes regulated by embryo sex in bovine morulae in a previous study (Denicol *et al*. 2015) as well as 24 additional genes.

**Figure 3:**
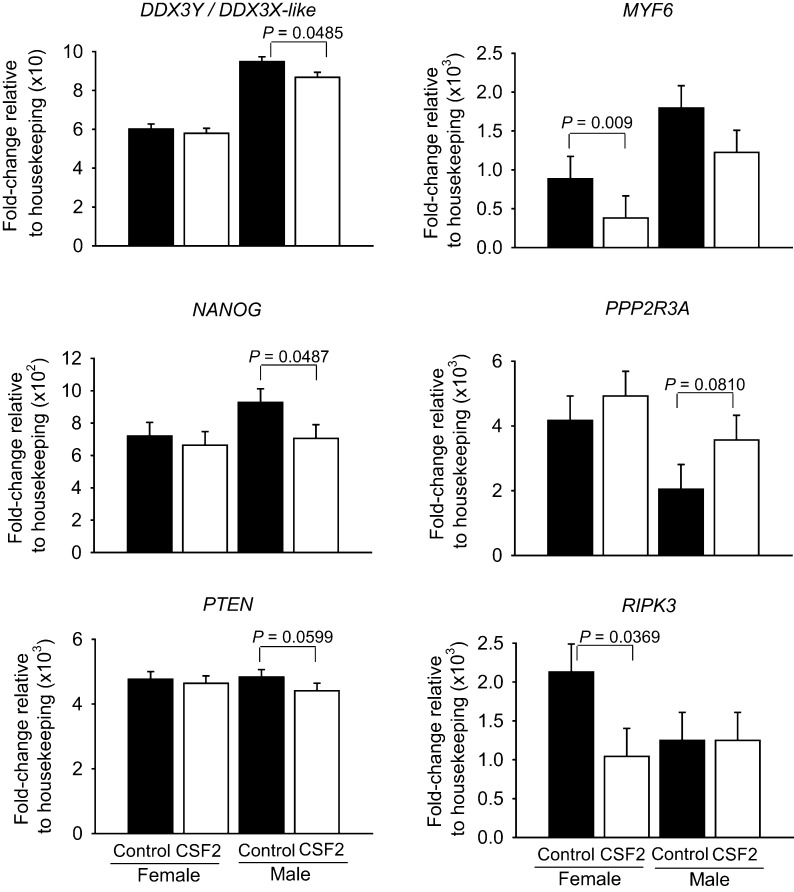
Genes affected by the main effect of CSF2. Data represent the fold change of level of expression relative to housekeeping genes. Fold change was multiplied by the factor shown on the *Y* axis for ease in graphing. Results are least-squares means ± s.e.m. of eight biological replicates of 50 pooled morulae each. *P* value for the main effects of sex and CSF2 treatment (trt) are shown for all effects where *P* < 0.10 or less. In addition, pairwise comparisons of effects of CSF2 were performed separately for female and male embryos and those effects where *P* was <0.10 or less are indicated by the brackets.

**Figure 4:**
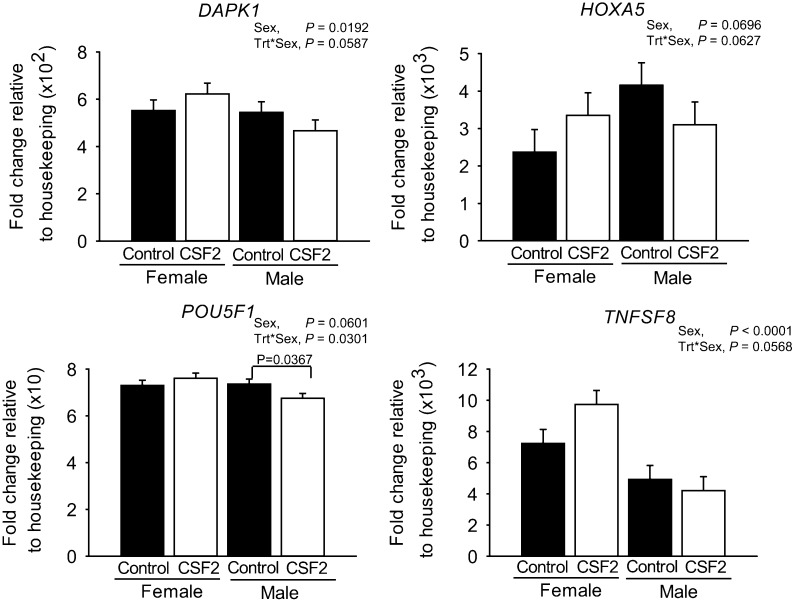
Genes affected by the interaction between CSF2 treatment and sex. Data represent the fold change of level of expression relative to housekeeping genes. Fold change was multiplied by the factor shown on the *Y* axis for ease in graphing. Results are least-squares means ± s.e.m. of eight biological replicates of 50 pooled morulae each. *P* value for the main effects of sex, CSF2 treatment (trt), and the interaction are shown for all effects where *P* < 0.10 or less. In addition, pairwise comparisons of effects of CSF2 were performed separately for female and male embryos and those effects where *P* was <0.10 or less are indicated by the brackets.

There were a total of 10 genes whose expression was affected by the main effect of CSF2 (Fig. 3) or the CSF2 by sex interaction (Fig. 4). Further evaluation of results indicated that, for each of these 10 genes, sex modified the changes in transcript abundance in response to CSF2. Either CSF2 affected expression for one sex only (*DDX3Y/DDX3X-like*, *P* = 0.0485; *MYF6*, *P* = 0.009; *NANOG*, *P* = 0.0487; *PPP2R3A*, *P* = 0.0810; *POU5F1*, *P* = 0.0301; *PTEN*, *P* = 0.0599; and *RIPK3*, *P* = 0.0369) or there was a statistical effect of the CSF2 × sex interaction (*DAPK1*, *P* = 0.0587; *HOXA5*, *P* = 0.0627; *POU5F1*, *P* = 0.0301; *TNFSF8*, *P* = 0.0568). As shown in Fig. 3, CSF2 decreased expression of *DDX3Y/DDX3X-like* (*P* = 0.0485), *NANOG* (*P* = 0.0487) and *PTEN* (*P* = 0.0599) in males but not females, increased expression of *PPP2R3A* in males (*P* = 0.0810) but not females, and decreased expression of *MYF6* (*P* = 0.009) and *RIPK3* (*P* = 0.0369) in females but not males. For three of the four genes affected by the interaction of CSF2 and sex (*DAPK1*, *HOXA5*, *POU5F1* and *TNFSF8*), CSF2 increased gene expression in females and decreased expression in males (Fig. 4). For *POU5F1*, CSF2 decreased expression in males and had no effect on females. Note that of the 22 genes upregulated in females, three were affected by CSF2 treatment in a sex-dependent manner (*DAPK1*, *PPP2R3A* and *TNFSF8*). Of the 11 genes upregulated in males, two were affected by CSF2 in one sex (*DDX3Y/DDX3X-like* and *MYF6*).

Note also that 6 of the 10 genes affected by CSF2 or CSF2 × sex were among a set of 48 genes that were earlier found to be modified by CSF2 at the morula stage in embryos produced with conventional semen (Loureiro *et al*. 2011). Of these genes, two (*PPP2R3A* and *RIPK3*) were affected in the same direction as found by Loureiro *et al*. (2011), one gene (*MYF6*) was affected in the opposite direction, and the direction of the effect was dependent on sex for three other genes (*DAPK1*, *HOXA5*, *TNFSF8*). A total of 42 of the 48 genes found earlier by (Loureiro *et al*. 2011) to be affected by CSF2 were not significantly affected in this experiment.

### Experiment 2: effects of CSF2 on development in female and male embryos

**Figure 5:**
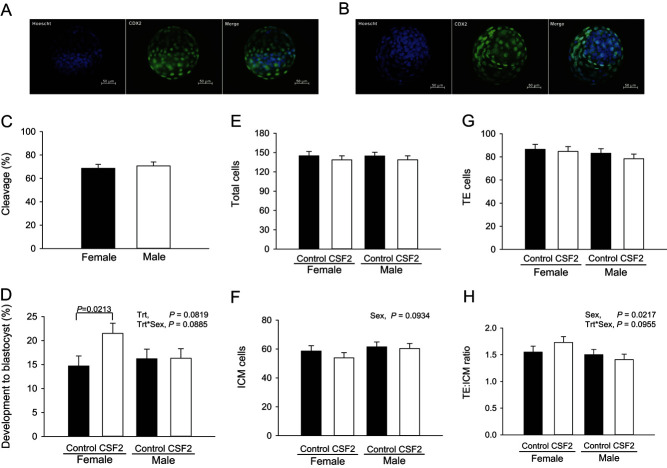
Effect of CSF2 and sex on cleavage and development of embryos to the blastocyst stage. The top panel shows representative images of a female (A) and a male (B) Day 7 blastocyst labeled with Hoechst (all nuclei) and anti-CDX2 (trophectoderm (TE) cells). The graphs represent least-squares means ± s.e.m. for cleavage (C), percent putative zygotes developing to the blastocyst stage (D), numbers of total (E), inner cell mass (ICM; F), trophectoderm (TE; G), and the ratio of TE to ICM cell numbers (H). The experiment was replicated nine times with a total of 351–427 putative zygotes per treatment group. *P* value for the main effects of sex, CSF2 treatment (trt) and the interaction are shown for all effects where *P* < 0.10 or less. In addition, pairwise comparisons of effects of CSF2 were performed separately for female and male embryos and those effects where *P* was <0.10 or less are indicated by the brackets.

There was no difference (*P* > 0.10) in the percent of putative zygotes that cleaved at Day 3 between oocytes fertilized with X- or Y-sorted semen (Fig. 5C). The percent of putative zygotes becoming blastocysts was affected by CSF2 (*P* = 0.0819) and the treatment × sex interaction (*P* = 0.0885; Fig. 5D). Similar results were observed for the percent of cleaved embryos becoming blastocysts (CSF2, *P* = 0.0810; results not shown). Further analysis using pairwise comparisons indicated that, among female embryos, CSF2 increased the proportion of putative zygotes (*P* = 0.0213) and cleaved embryos (*P* = 0.0252) that became blastocysts at Day 7 compared with controls. On the contrary, CSF2 had no effect on development of male embryos to the blastocyst stage (Fig. 5D).

Overall, there was no effect of sex on total number of blastomeres or number of TE cells in the blastocyst (Fig. 5E and G). Female embryos tended to have a lower number of ICM cells compared with males (56.2 ± 3.1 vs 61.0 ± 2.9 respectively; *P* = 0.0934; Fig. 5F) and, consequently, TE:ICM ratio was higher in female blastocysts (1.64 ± 0.09 vs 1.45 ± 0.08 respectively; *P* = 0.0217; Fig. 5H). There were no effects of CSF2 or CSF2 × sex on total number of cells or on number of TE or ICM cells.

## Discussion

In recent years, evidence has accumulated to support the idea that sex plays a major role in determining molecular and cellular responses of the embryo to its environment as early as the preimplantation period (Watkins *et al*. 2008, Dobbs *et al*. 2014, Donjacour *et al*. 2014, Denicol *et al*. 2015, Lowe *et al*. 2015, Hansen *et al*. 2016). One possible explanation for differences in responses of female and male embryos to changes in maternal environment is that maternal embryokines that undergo a change in secretion in response to environmental stimuli act on female embryos differently than on male embryos. A candidate for mediating changes in maternal environment on embryonic development is CSF2. Maternal secretion of this cytokine into the reproductive tract can be altered by exposure to semen (Tremellen *et al*. 1998, O’Leary *et al*. 2004, Bromfield *et al*. 2014) and obesity (Nahar *et al*. 2013). Moreover, CSF2 exerts a variety of actions on embryonic development (Sjöblom *et al*. 1999, 2005, Loureiro *et al*. 2009, 2011, Kwak *et al*. 2012) that result in increased competence to establish pregnancy after transfer into female recipients (Loureiro *et al*. 2009, Ziebe *et al*. 2013, Denicol *et al*. 2014). In this paper, we provide data that demonstrates that, in the cow, CSF2 affects embryonic development and gene expression in a sex-dependent manner. In particular, CSF2 affected gene expression in the morula differently for female and male embryos and also improved the competence of putative zygotes to develop to the blastocyst stage only in females. Along with previous findings that CSF2 treatment during Day 5–7 of development affects trophoblast elongation at Day 15 differently in female and male embryos (Dobbs *et al*. 2014), these results mean that CSF2 is a maternally derived molecule that regulates embryonic development in a sex-specific manner.

It is not surprising that sex would modify responses of the preimplantation embryo to regulatory signals because female and male embryos are different from each other as early as the morula stage, when embryos in this experiment were treated with CSF2. Expression of a total of 33 of the 90 genes examined was affected by sex. In fact, differences in gene expression between females and males is most probably greater than that observed here because use of X- and Y-sorted sperm to produce male and female embryos means that about 10% of the embryos are of the non-desired sex (DeJarnette *et al*. 2009). By the blastocyst stage, one-third of expressed genes are differentially expressed between female and male embryos (Bermejo-Alvarez *et al*. 2010). Many of the genes found to be regulated by sex in the present experiment play important roles in cellular function and could, therefore, change the regulatory network within embryonic cells so that responses to CSF2 are different for female and male embryos. One gene upregulated in female embryos was *XIST*, which encodes for a non-coding RNA responsible for X chromosome inactivation (Huynh & Lee 2005), which in turn is involved in control of WNT signaling, pluripotency and differentiation, and DNA methylation (Sato *et al*. 2004, Schulz *et al*. 2014, Madeja *et al*. 2015, Gayen *et al*. 2016). Skewing of sex ratio in mouse embryos produced in vitro has been ascribed to epigenetic effects on *XIST* (Tan *et al*. 2016). Among the other genes regulated by sex are those encoding for transcriptional regulators (*FHL1*, *KLF8*, *PEG3* and *ZNF180* upregulated in females and *CREM* and *MYF6* upregulated in males), translation factors, regulatory ligands, receptors and intracellular signaling proteins (*PPP2R3A*, *RNF7*, *TGFB1* and *TNFSF8* upregulated in females and *BMP15*, *DDX3Y/DDX3X-like*, *EIF1AY*, *FGF4* and *NOTCH2* upregulated in males) and proteins involved in epigenetic remodeling (*DNMT3A* and *PRMT1* upregulated in males). Consistent with the recent observation that female embryos are more prone to apoptosis in cattle than male embryos (Ghys *et al*. 2016), several proapoptotic genes were upregulated in female embryos (*APOPT1*, *BAK1*, *DAPK1* and *TNFSF8*). One of the genes upregulated in females promotes pluripotency of ICM (*AMOT;* Leung & Zernicka-Goetz 2013), while several genes upregulated in males promote differentiation of one or cell lineages including *CDX2* (TE, Goissis & Cibelli 2014), *FGF4* (hypoblast; Kuijk *et al*. 2012) and *MYF6* (muscle; Maak *et al*. 2006).

It has long been known that CSF2 can increase the proportion of cultured embryos that develop to the blastocyst stage in several species including cattle (Moraes & Hansen 1997, Loureiro *et al*. 2009). In this species, however, the effect of CSF2 has been reported to vary with the overall level of development of cultured embryos. When development was low, CSF2 increased the percent of embryos that became blastocysts whereas, when development was high, CSF2 decreased development to the blastocyst stage (Dobbs *et al*. 2013*b*). The present finding that CSF2 increased the competence of embryos to become blastocysts only when embryos were females could possibly explain some of this phenomenon because, in cattle, male embryos have been reported to have greater competence to develop to the blastocyst stage in culture than female embryos, at least in some culture media (Green *et al*. 2016).

Sexual dimorphism in CSF2-induced changes in gene expression could explain in part why CSF2 increased blastocyst development in females but not males. In females, CSF2 increased expression of *POU5F1*, a transcription factor involved in pluripotency (Nichols *et al*. 1998, Radzisheuskaya & Silva 2014) and *HOXA5*, a transcriptional regulator of developmental processes and cell positional identities (Chen *et al*. 2005). Also, CSF2 decreased the proapoptotic gene *RIPK3* which could presumably increase cell number in the developing embryo. For male embryos, with the exception of *RIPK3*, expression of each of these genes was either not affected or was altered by CSF2 in the opposite direction. Moreover, CSF2 increased expression of the antiproliferative gene *PPP2R3A* (Kurimchak & Graña 2015) in male embryos only. CSF2 also had a greater inhibitory effect on expression of the myogenic transcription factor *MYF6* in female embryos than in male embryos. Actions on expression of *POU5F1* and *MYF6* could conceivably promote pluripotency in female embryos.

One surprising feature of the results was that a large number of genes previously reported to be regulated by CSF2 in bovine morulae produced with a mixture of X- and Y-bearing spermatozoa (Loureiro *et al*. 2011) were not significantly affected by CSF2 in this experiment. It is noteworthy that the experiment of Loureiro *et al*. (2011) involved microarray analysis, which can be difficult to replicate (Weis 2005, Shi *et al*. 2008). It was also surprising that the X-linked gene *BMP15* was expressed to a greater degree in males compared with females. This sex effect on *BMP15* expression was not observed earlier (Denicol *et al*. 2015) and may be an anomaly. This is unlikely, however, because we have also observed higher expression of *BMP15* in male blastocysts than female blastocysts (Moss, Tribulo and Hansen, unpublished). Perhaps *BMP15* transcription is reduced in females despite the presence of two X chromosomes because of epigenetic differences between sexes or mRNA stability for *BMP15* is lower in females.

A total of four of the genes studied are subject to imprinting – *H19*, *IGF2*, *PEG3* – and *XIST*. Of these, expression was higher in females for two genes (*PEG3* and *XIST*) but there were no effects of CSF2 or interactions of CSF2 with sex. A broader analysis of the effects of CSF2 and sex on imprinted genes is warranted to determine whether imprinted genes are regulated preferentially by sex or CSF2.

In conclusion, CSF2 treatment during culture exerted divergent actions on gene expression and development of female and male preimplantation embryos. Several genes important for development or other aspects of cellular function were regulated differently in female and male embryos and CSF2 increased competence to develop to the blastocyst stage for female embryos only. Thus, CSF2 may be involved in sexually dimorphic responses of embryos to changes in maternal environment. Further research is required to understand actions of CSF2 on allocation of cells in the blastocyst to ICM and TE and to understand how actions of CSF2 on the preimplantation embryo affect survival after transfer to recipients. At least for cattle, experiments showing CSF2 improved embryonic survival after transfer to cows was based on embryos produced with X-sorted semen (Loureiro *et al*. 2009, Denicol *et al*. 2014). It remains to be determined whether a similar effect of CSF2 occurs for male embryos.

## Supplementary data

This is linked to the online version of the paper at http://dx.doi.org/10.1530/REP-16-0336.

## Declaration of interest

The authors declare that there is no conflict of interest that could be perceived as prejudicing the impartiality of the research reported.

## Funding

This research was supported by NIH Grant R03 HD080855.
